# Study of the Association between Thiols and Oxidative Stress Markers in Children with Obesity

**DOI:** 10.3390/nu14173637

**Published:** 2022-09-02

**Authors:** Annamaria D’Alessandro, Giovina Di Felice, Melania Manco, Anna Pastore, Simona Pezzi, Michela Mariani, Danilo Fintini, Andrea Onetti Muda, Ottavia Porzio

**Affiliations:** 1 Clinical Biochemistry Laboratory, IRCCS “Bambino Gesù” Children’s Hospital, 00165 Rome, Italy; 2 Research Unit of Multifactorial and Complexes Phenotype Diseases, IRCCS “Bambino Gesù” Children’s Hospital, 00165 Rome, Italy; 3 Research Unit of Diagnostical and Management Innovations, IRCCS “Bambino Gesù” Children’s Hospital, 00165 Rome, Italy; 4 Endocrinology Unit, University Pediatric Clinical Department, IRCCS “Bambino Gesù” Children’s Hospital, 00165 Rome, Italy; 5 Department of Experimental Medicine, Tor Vergata University, 00133 Rome, Italy

**Keywords:** obesity, oxidative stress, thiols, glutathione

## Abstract

Obesity has reached epidemic proportions, and the World Health Organization defined childhood overweight and obesity as a noncommunicable disease that represents the most serious public health challenges of the twenty-first century. Oxidative stress, defined as an imbalance between oxidants and antioxidants causing an impairment of the redox signals, is linked to the development of metabolic diseases. In addition, reactive oxygen species generated during metabolic disorder could increase inflammation, causing the development of insulin resistance, diabetes, and cardiovascular disease. We analyze serum levels of cysteine (Cys), cysteinyl-glycine (Cys-Gly), homocysteine (Hcy), and glutathione (GSH), and other markers of oxidative stress, such as thiobarbituric acid reactive substances (T-BARS), 8-isoprostane, and protein carbonyl in our children with obesity. Total antioxidant status was also determined. We found lower GSH and Cys-Gly levels, and higher Hcy and oxidative stress markers levels. We also found a positive correlation between Body Mass Index (BMI), Cys, GSH, and Hcy levels, between insulin and Cys levels, and between BMI and the homeostasis model assessment-estimated insulin resistance (HOMA-IR) with 8-isoprostane levels. Finally, we found a correlation between age and GSH and Cys levels. The deficiency of GSH could be restored by dietary supplementation with GSH precursors, supplying an inexpensive approach to oppose oxidative stress, thus avoiding obesity complications.

## 1. Introduction

Disruption of normal metabolic processes could lead to redox imbalance and the initiation of several pathophysiological changes collectively termed metabolic syndrome, caused by overweight and obesity. Obesity has reached epidemic proportions, and the World Health Organization defined childhood overweight and obesity as a noncommunicable disease that represents the most serious public health challenges of the twenty-first century [1]. Indeed, in the last 30 years, childhood obesity has increased constantly in both low- and high-income countries [2,3,4]. Obesity in children and adolescents is defined as a BMI greater than or equal to the 95th percentile by age and sex of the child or a BMI of more than 30.0 kg/m^2^ among older adolescents (that is the definition of obesity in adults).

Oxidative stress, defined as an imbalance between oxidants and antioxidants causing an impairment of the redox signals, is related to chronic inflammation and to the evolution of metabolic diseases [5]. Oxidative stress is higher in children with obesity, where it could boost the evolution of complications [6,7]. Insulin resistance is frequently linked to obesity, and it could contribute as a fount of oxidative stress [8]. Additionally, reactive oxygen species (ROS) generated during metabolic disorder could increase inflammation by damaging redox signaling pathways and modifying gene expression of inflammatory cytokines, chemokines, and growth factors, thus causing the progress of several diseases, such as insulin resistance, diabetes, and cardiovascular disease [9].

As several studies have highlighted the clinical impact of thiol-disulfide hemostasis on obesity [10,11,12,13,14,15], we analyze thiol levels in our cohort of children with obesity. Moreover, we investigate serum markers of oxidative stress in order to verify if ROS overproduction could contribute to the development of complications in obesity. We thus determine the serum levels of cysteine (Cys), cysteinyl-glycine (Cys-Gly), homocysteine (Hcy), and glutathione (GSH), and other markers of oxidative stress, such as thiobarbituric acid reactive substances (T-BARS), 8-isoprostane, and protein carbonyl. Moreover, we also analyze the total antioxidant, as Trolox equivalents, in our cohort of children with obesity.

## 2. Materials and Methods

### 2.1. Study Type

This retrospective case–control study includes 283 consecutive patients with BMI between 26 and 40.0 kg/m^2^ and 105 normal-weight sex-matched children enrolled in the Nutritional Education Program of the Bambino Gesù Children’s Hospital and Research Institute of Rome, Italy, from October 2020 and February 2022.

Inclusion criteria were:

- Written informed consent of the subjects legally authorized to give consent or the parent(s)/legal representative of the minors according to national law;
- Consent in school-age subjects, i.e., aged ≥ 6 years;
- Male and/or female subjects aged between 2 and 18 years;
- Caucasian race;
- Overweight (i.e., a BMI ≥ 85th (1036 standard deviation score, SDS) and < 95th percentile (1645 SDS)) according to the Italian growth curves;
- Non-syndromic obesity (i.e., a BMI ≥ 95th percentile; BMI z-score for age and sex < 1064 SDS);
- Absence of systemic/endocrine pathology (except overweight/obesity).

Exclusion criteria were as follows:

- No written informed consent of the patient or parents or no consent of the minor if applicable.
- Genetic obesity and other genetically determined syndromes;
- Any condition associated with an increase in inflammatory parameters and, specifically, with values of erythrocyte sedimentation rate (ESR) > 15 mm or with C-reactive protein (CRP) > 0.50 mg/dL, or with a number of white blood cells > 16.00 × 10^3^/µL.
- Presence of cognitive deficits;
- Presence of previous thromboembolic or hemorrhagic events;
- Presence of anticoagulant therapy;
- Presence of chronic corticosteroid therapy;
- Positive lupus anticoagulant (LA) test;
- Bariatric therapy;
- Presence of allergies (total immunoglobulin E (IgE) ≥ 100.0 kU/L).

Controls inclusion criteria were:

- Written informed consent of the subjects legally authorized to give consent or the parent (s)/legal representative of the minors according to national law;
- Consent in school-age subjects, i.e., aged ≥ 6 years;
- Male and/or female subjects aged between 2 and 18 years;
- Caucasian race;
- Normal weight (BMI z-score for age and sex < 1.064 SDS), that is a body mass index between the 10th and 84th percentiles;

Exclusion criteria for controls were:

- No written informed consent of the patient or parents or no consent of the minor if applicable;
- Presence of inflammatory processes in the lower or upper respiratory tract, erythrocyte sedimentation rate values (ESR) ≥ 15 mm, C-Reactive Protein (CRP) ≥ 0.50 mg/dL, number of white blood cells ≥16.00 × 10^3^/µL;
- Suspected celiac disease (anti-transglutaminase antibodies ≥ 20 CU);
- Presence of chronic corticosteroid therapy;
- Presence of cognitive deficits;
- Presence of previous thromboembolic or hemorrhagic events;
- Presence of anticoagulant therapy;
- Positive lupus anticoagulant (LA) test;
- Presence of allergies (total IgE ≥ 100.0 kU/L).

### 2.2. Samples

Blood was collected into Vacutainer Tubes (Becton Dickinson, Franklin Lakes, NJ, USA); plasma and/or serum was obtained immediately by the centrifugation of the blood at 2000 g for 5 min. and stored at −80 °C until analysis within a maximum of one month from the collection. Technical staff collected clinical data and informed consent from participants, and the corresponding samples were labeled and analyzed as described below. To maintain confidentiality, participants were labeled with de-identified barcodes, and all data were collected and stored in a locked room with limited access.

### 2.3. Thiol Determination

Cysteine (Cys), cysteinyl-glycine (Cys-Gly), homocysteine (Hcy), and glutathione (GSH) were analyzed as previously reported [16]. We updated the chromatographic system, thus the high-performance liquid chromatography (HPLC) was an Agilent Technologies 1290 Infinity System (Agilent Technologies, Waldbronn, Germany, EU) furnished with a fluorescence detector operating at an excitation wavelength of 390 nm and an emission wavelength of 478 nm. The signals acquired were examined by the Agilent OpenLab CDS ChemStation Edition (software vA.02.15; Agilent Technologies, Waldbronn, Germany, EU). Column (Hypersil ODS; 150 × 4.6 mm, 3 μm particle size) and precolumn (Hypersil ODS; 10 × 4 mm, 5 μm particle size) were supplied by Thermo Scientific (Thermo Fisher Scientific, Bellefonte, PA, USA).

### 2.4. Total Antioxidant Levels and Oxidative Stress Markers

Total antioxidant, thiobarbituric acid reactive substances (T-BARS), 8-isoprostane, and protein carbonyl levels were analyzed by a commercial kit following the manufacturer’s instructions (Cayman Chemical, Ann Arbor, MI, USA).

### 2.5. Other Laboratory Assays

Biochemical parameters, such as glucose, insulin, C-reactive protein (CRP), total cholesterol, low-density lipoprotein (LDL), high-density lipoprotein (HDL), triglycerides, alanine aminotransferase (ALT), and aspartate aminotransferase (AST), were measured on samples collected upon admission by using the Roche Cobas 8000/c702 module (Roche Diagnostics, Basel, Switzerland). Insulin and glucose levels were used for the calculation of the Homeostatic Model Assessment for Insulin Resistance (HOMA-IR) [17].

### 2.6. Statistical Analysis

Statistical analysis was performed using GraphPad Prism v9.0 (GraphPad Software, San Diego, CA, USA). To test for normal distribution of data, both the histogram and the Kolmogorov–Smirnov test of normality were used. If the data were normally distributed, parametric tests such as analysis of variance with Bonferroni post hoc test (in the case of more than two variables), or *t*-test (in the case of two variables) were used. In order to test the differences between different groups, the Mann–Whitney U test was used. A value of *p* < 0.05 was considered statistically significant in all statistical analyses; a value of *p* < 0.01 was considered extremely statistically significant. Spearman’s rank correlation between the features extracted from each patient was also performed.

## 3. Results

### 3.1. Characteristics of the Patients Studied

**Figure 1:**
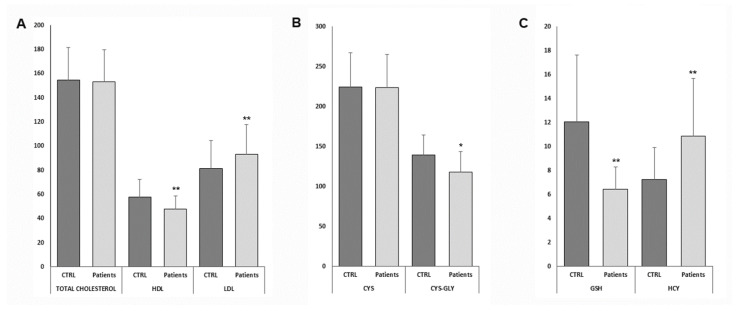
Comparisons of cholesterol and thiol levels. (**A**) Concentrations of Total, LDL and HDL cholesterol, expressed as mg/dL. (**B**) Cysteine (Cys) and cysteinyl-glycine (Cys-Gly) levels. (**C**) Concentrations of homocysteine (Hcy), and glutathione (GSH). Thiol levels are expressed as μmol/L. * *p* < 0.05; ** *p* < 0.01. All parameters are presented as mean ± standard deviation. Abbreviations: controls (CTRL); high-density lipoproteins (HDL); low-density lipoproteins (LDL).

In Table 1 are summarized the demographic and clinical data of the patients studied. The mean BMI value in our population is 32.41 (±7.19), with a mean age of about 12.11 years (±5.69). Regarding patients’ gender, we report the separate values found in male and female patients. No significant differences were found, except for age and AST and ALT values, demonstrating the absence of a gender-related condition. Median CRP levels were higher in our patients when compared to reference values (0.25 vs. <0.05 mg/dL, respectively; *p* < 0.01), consistent with previous findings correlating obesity with inflammation also in our cohort [18]. Regarding cholesterol, although the total levels were between the reference values for the pediatric population (153.39 ± 26.19 vs. 154.68 ± 12.54 mg/dL; *p* = 0.40), the LDL form is significantly higher (93.33 ± 24.60 vs. 81.21 ± 18.53 mg/dL; *p* < 0.01) and HDL lower (47.87 ± 10.75 vs. 58.00 ± 8.22 mg/dL; *p* < 0.01) compared to pediatric reference values (Figure 1A). These results sustain the link between obesity and cardiovascular risk through increased fasting plasma LDL cholesterol, low HDL cholesterol, elevated blood glucose, and insulin levels risk factors [19]. Triglycerides are also higher in patients with obesity with respect to controls (92.80 ± 48.92 vs. 61.25 ± 21.13 mg/dL: *p* < 0.01). In addition, we also found elevated HOMA-IR values compared to those reported for pediatric subjects without increased metabolic risk (4.83 ± 2.9 vs. 3.20 ± 0.24; *p* < 0.05) [20], confirming obesity as a risk factor for metabolic disorders [21].

### 3.2. Thiol Status

Table 1 showed also the thiol status of our patients. Here we report an imbalance of the glutathione metabolism parameter. In particular, Cys-Gly (an intermediate of the glutathione synthesis) is lower in patients with obesity with respect to controls (125.80 ± 25.60 vs. 139.90 ± 25.96 μmol/L; *p* < 0.05). Accordingly, the product of the glutathione cycle (GSH) is also significantly decreased in patients with respect to healthy subjects (6.06 ± 2.45 vs. 2.16 ± 5.70 μmol/L; *p* < 0.01). In addition, Hcy levels were also significantly increased (10.00 ± 4.43 vs. 7.29 ± 2.66 μmol/L; *p* < 0.01). In Figure 1 are summarized the comparisons of some of the results reported in Table 1 between patients and controls.

### 3.3. Oxidative Stress Parameter and Antioxidant Status

**Figure 2:**
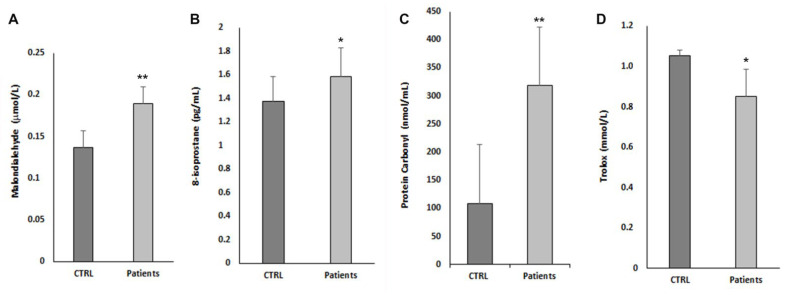
Oxidative stress parameter and antioxidant levels. (**A**) T-BARS levels, expressed as malondialdehyde (MDA) equivalent; (**B**) 8-isoprostane levels; (**C**) concentrations of protein carbonyl. Total antioxidants levels, expressed as Trolox equivalents, are reported in (**D**). * *p* < 0.05; ** *p* < 0.01. All parameters are presented as mean ± standard deviation.

Figure 2 reported the oxidative stress parameter studied in patients with obesity. In particular, all the markers of oxidative stress, such as T-BARS, expressed as malondialdehyde (MDA) equivalent (0.19 ± 0.02 vs. 0.14 ± 0.02 μmol/L; *p* < 0.01), 8-isoprostane (1.58 ± 0.24 vs. 0.38 ± 0.21 pg/mL; *p* < 0.05), and protein carbonyl (317.94 ± 59.62 vs. 107.58 ± 9.42 nmol/mL; *p* < 0.01) are significantly higher in subjects with obesity. In addition, Total Antioxidants levels, expressed as Trolox equivalents, are lower than in controls (0.85 ± 0.13 vs. 1.05 ± 0.03 nmol/L; *p* < 0.01).

### 3.4. Correlations between Thiols, Oxidative Stress, and the Other Parameters Studied

**Figure 3:**
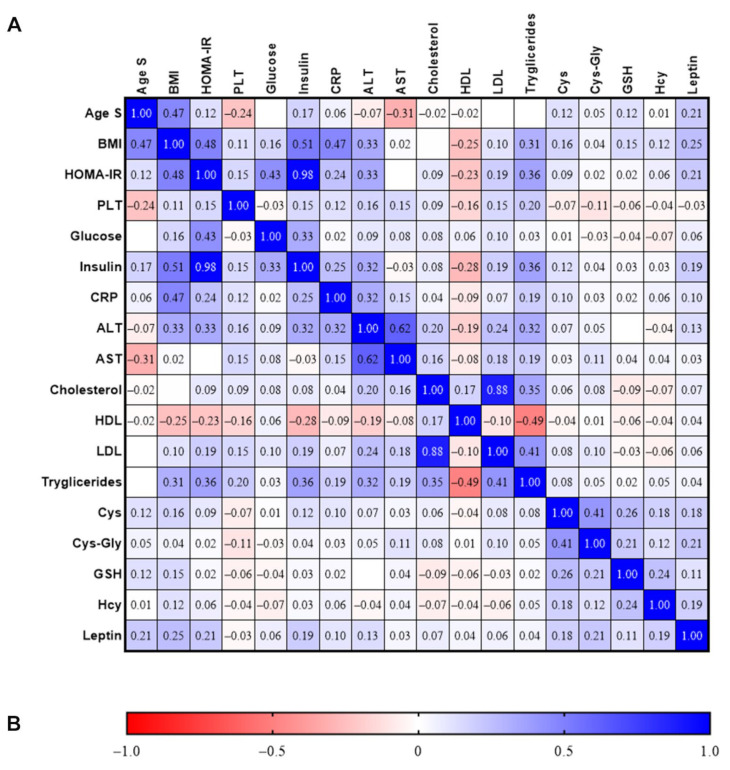
Correlations between thiol levels, oxidative stress parameters, antioxidant levels, and the other parameters studied. Values are expressed as Spearman correlation coefficient (*r*) (**A**). Color explanation is reported in (**B**), with the scale ranging from negative correlation (red) to positive correlation (blue). White box indicates no correlation.

The correlations between the various parameters studied are reported in Figure 3. A positive correlation was determined between BMI, Cys, GSH, and Hcy levels (*r* = 0.163, *p* < 0.01, *r* = 0.146, *p* < 0.05, and *r* = 0.116, *p* < 0.01, respectively), and between insulin and Cys levels (*r* = 0.118, *p* < 0.05). Regarding the other oxidative stress parameters, no correlation was found, except for a positive correlation between BMI and 8-isoprostane levels (*r* = 0.368, *p* < 0.05) and HOMA-IR and 8-isoprostane levels (*r* = 0.402, *p* < 0.05). Regarding patient’s age, we found a positive correlation with GSH levels (*r* = 0.121, *p* < 0.05), Cys (*r* = 0.115, *p* < 0.05), BMI (*r* = 0.470, *p* < 0.01), insulin (*r* = 0.169, *p* < 0.01), and HOMA-IR (*r* = 0.123, *p* < 0.05), and a negative correlation with AST (*r* = −0.313, *p* < 0.01). No correlation was found between the other parameters studied and thiol and oxidative stress levels (*p* > 0.05).

## 4. Discussion

The existing correlation between inflammation and obesity has been demonstrated [18]. Consistent with these previous findings, we found that CRP levels were significantly higher in our children with obesity. In addition, we also found elevated HOMA-IR values compared to those reported for pediatric subjects without increased metabolic risk, confirming that obesity is a risk factor for metabolic disorders [21]. Moreover, we found high LDL and low HDL cholesterol levels. These results support the link between obesity and cardiovascular risk through increased fasting plasma LDL cholesterol, low HDL cholesterol, and elevated blood glucose and insulin levels [19].

Recently, it was demonstrated that the thiol/disulfide homeostasis antioxidant parameters were lower in obese children, with consequent higher oxidant parameters and a shift of the thiol/disulfide balance toward disulfide formation [10]. These findings do not corroborate in adult women with obesity, in which the oxidative damage is demonstrated only in plasma proteins with antioxidant function [11]. Thiol unbalance could thus be an early event culminating in the loss of function of antioxidant plasma proteins, probably due to their irreversible oxidation caused by the cumulating ROS in the environment.

Several studies have highlighted the clinical impact of homocysteine on obesity [12,13,14,15]. This is corroborated in this study, as we found that Hcy is significantly higher in patients with obesity with respect to healthy children. It was extensively demonstrated that high Hcy levels, which are correlated with lower HDL cholesterol in blood in volunteers and animal models, have been associated with an increased risk for cardiovascular diseases [22]. In humans, the relation between methionine and Hcy is dependent on vitamins B6-B12 and folic acid status and on the supply of other amino acids. However, lowering Hcy by itself is not sufficient for decreasing the risk of cardiovascular disease progression [23], as several other mechanisms are involved. First, the connection of one carbon metabolism and protein biosynthesis editing mechanisms has been demonstrated. Thus, Hcy and its metabolites could affect epigenetic control of gene expression, which underlies the pathology of several human diseases [24]. Therefore, Hcy is the hallmark of this imbalance [25]. Second, a decrease in the concentration of intracellular glutathione, a tripeptide involved in redox homeostasis, is also implicated [26]. Glutathione has indeed a fundamental role in the protection from free radicals, and its reduction could cause a chain of dangerous reactions eventually terminating in oxidative stress and presumably in metabolic syndrome [27]. Glutathione reduction could be caused by a reduced glutathione synthesis, an augmented utilization, or a coalescence of both mechanisms. Accordingly, we found dramatically lower levels of GSH in our children with obesity. Our previous studies report an excess of GSH utilization in obesity [14,15], and Nguyen and collaborators [28] suggest that GSH deficiency can promote fat accumulation. They found that mice with GSH deficiency had significantly higher total body fat, and that restoring GSH led to significant fat reductions, suggesting a role for GSH in maintaining fat homeostasis. Consistent with these findings, Chen and colleagues [29] showed that genetic manipulations induce severe hepatic depletion of GSH, resulting in excessive accumulation of fat in the liver of mice, thus suggesting that GSH deficiency could promote fat accumulation. In agreement with these findings, we showed a 50% decrement of GSH levels in the serum of our patients with obesity compared to our control population. This result agrees with our previous findings in the blood of children with obesity, in which we evidenced a depletion of Tot GSH, which is indicative of a defect in the thiol synthesis [14]. This is sustained by the fact that the significantly lower levels of Cys-Gly, an intermediate of the GSH synthesis, are not offset by an equivalent rise of Cys. This could be due to the impaired glycine status found in children with obesity [30]. This finding is also corroborated by Okekunle and colleagues [31], who demonstrated that plasma glycine levels are steadily lower in patients with obesity and T2DM compared to controls (−11% and −15%, respectively). It was earlier demonstrated that glycine availability may be the limiting factor for glutathione synthesis [32]. Indeed, glycine levels in tissues are lower than the Michaelis–Menten constant (Km) of glutathione synthase so that the accessibility of glycine could be excessively low to sustain a satisfactory synthesis rate of GSH. This is particularly true in metabolic diseases, in which oxidative stress is increased [33]. It has been hypothesized that oxidative stress, defined as the imbalance between oxidants and antioxidants with an increase of oxidants, could impair intracellular redox signals, leading to chronic inflammation and the development of metabolic diseases [5]. The involvement of oxidative stress in the development of obesity in adults has been extensively studied [34], but less information is available in children. Here we demonstrated a rise in oxidative stress parameter in children with obesity, along with lower total antioxidants. Accordingly, enhanced ROS formation and the increase in oxidatively modified proteins have been previously demonstrated in both overweight and obese youths, and have been implicated in the progress of obesity-related disorders [35].

The deficiency of GSH and the gained oxidative stress evidenced by our study may have cumulative effects; indeed, low GSH levels could intensify the oxidative damage, either of its unavailability as a direct scavenger of free radicals, or of its function as a cofactor for the antioxidant glutathione-dependent enzymes (glutathione peroxidase (GPx), glutathione transferase (GST), and glutathione reductase (GR)). However, as also sustained by the latest literature [34,35,36,37,38,39], glutathione and glycine reduction could be repaired by dietary supplementation with precursors, thus allowing a reasonable approach to neutralize oxidative stress and prevent obesity complications. Furthermore, it was recently demonstrated that diet could increase serum concentrations of chronic inflammation and oxidative stress markers. Early dietary interventions and supplementations could thus decrease chronic inflammation and oxidative stress in children and adolescents [40].

## Figures and Tables

**Table 1:** Clinical and demographic characteristics of our patients.

Variable	All Patients	Male	Female	*p*
*n*	283	162	121	
Age, y, mean (standard deviation)	12.11 (5.69)	12.04 (2.89)	12.85 (3.47)	0.037
Height, cm, mean (standard deviation)	154.28 (14.62)	77.37 (25.44)	74.97 (23.67)	0.16
Weight, Kg, mean (standard deviation)	76.30 (24.66)	155.30 (15.28)	153.02 (13.72)	0.39
BMI, mean (standard deviation)	32.41 (7.19)	31.01 (6.20)	31.41 (3.47)	0.59
CRP, mg/dL, median (25th and 75th percentile)	0.20 (0.07–0.42)	0.21 (0.07–0.41)	0.19 (0.07–0.42)	0.46
HOMA-IR, median (25th and 75th percentile)	4.00 (2.22–5.97)	4.95 (2.27–5.72)	4.70 (2.80–5.70)	0.90
Triglycerides, mg/dL, mean (standard deviation)	89.83 (42.92)	96.30 (56.65)	88.19 (36.30)	0.14
Cholesterol, mg/dL, mean (standard deviation)	155.80 (31.91)	152.10 (24.73)	155.10 (28.11)	0.35
HDL, mg/dL, mean (standard deviation)	46.00 (40.00–53.00)	47.50 (10.54)	48.36 (10.98)	0.51
LDL, mg/dL, mean (standard deviation)	92.00 (75.00–108.50)	92.35 (22.94)	94.62 (26.78)	0.45
AST, U/L, median (25th and 75th percentile)	20.00 (16.00–27.25)	22.00 (19.00–28.00)	19.00 (16.00–24.00)	0.0002
ALT, U/L, median (25th and 75th percentile)	19.00 (15.00–29.00)	22.00 (16.00–34.00)	16.00 (13.00–23.00)	0.0006
Cys, μmol/L, mean (standard deviation)	223.30 (41.83)	221.00 (40.28)	227.00 (42.44)	0.23
Cys-Gly, μmol/L, mean (standard deviation)	125.80 (25.60)	119.20 (25.15)	116.50 (25.95)	0.37
GSH, μmol/L, mean (standard deviation)	6.06 (2.45)	6.26 (1.65)	6.65 (2.08)	0.09
Hcy, μmol/L, mean (standard deviation)	10.00 (4.43)	10.80 (5.12)	10.95 (4.34)	0.79

Abbreviations: Body Mass Index (BMI); C-reactive protein (CRP); homeostasis model assessment-estimated insulin resistance (HOMA-IR); high-density lipoproteins (HDL); low-density lipoproteins (LDL); aspartate aminotransferase (AST); alanine aminotransferase (ALT); cysteine (Cys); cysteinyl-glycine (Cys-Gly); glutathione (GSH); homocysteine (Hcy).

## Data Availability

The authors confirm that the data supporting the findings of this study are available within the article. Raw data that support the findings of this study are available from the corresponding author, upon reasonable request. The data are not publicly available due to privacy and ethical reasons.
